# Sea Ice Properties in High‐Resolution Sea Ice Models

**DOI:** 10.1029/2020JC016686

**Published:** 2021-01-13

**Authors:** Jinlun Zhang

**Affiliations:** ^1^ Polar Science Center Applied Physics Laboratory University of Washington Seattle WA USA

**Keywords:** Arctic, high‐resolution modeling, sea ice, sea ice rheology, sea ice strength parameter

## Abstract

An Arctic sea ice‐ocean model is run with three uniform horizontal resolutions (6, 4, and 2 km) and identical sea ice and ocean model parameterizations, including an isotropic viscous‐plastic sea ice rheology, a mechanical ice strength parameterization, and an ice ridging parameterization. Driven by the same atmospheric forcing, the three model versions all produce similar spatial patterns and temporal variations of ice thickness and motion fields, resulting in almost identical magnitude and seasonal evolution of total ice volume and mean ice concentration, ice speed, and fractions of ice of various thickness categories over the Arctic Ocean. Increasing model resolution from 6 to 2 km does not significantly improve model performance when compared to NASA IceBridge ice thickness observations. This suggests that the large‐scale sea ice properties of the model are insensitive to varying high resolutions within the multifloe scale (2–10 km), and it may be unnecessary to adjust model parameters constantly with increasingly high resolutions. This is also true with models within the aggregate scale (10–75 km), indicating that model parameters used at coarse resolution may be used at high or multiscale resolution. However, even though the three versions all yield similar mean state of sea ice, they differ in representing anisotropic properties of sea ice. While they produce a basic pattern of major sea ice leads similar to satellite observations, their leads are distributed differently in space and time. Without changing model parameters and sea ice spatiotemporal variability, the 2‐km resolution model tends to capture more leads than the other two models.

## Key Points

- Arctic sea ice models of varying high resolutions (2, 4, and 6 km) produce similar and realistic sea ice thickness without parameter changes
- Model parameters used at coarse resolution may be used at high resolution without a need to change
- The high‐resolution models all produce a basic pattern of major ice leads similar to satellite observations; the 2 km‐resolution model creates more leads

## 1 Introduction

Sea ice is an important component of the Earth’s climate system. The sea ice cover is highly heterogeneous and discontinuous. In the marginal ice zone, there are low concentration, low thickness floes of varying sizes and shapes (Rothrock & Thorndike, 1984). In the ice pack interior, a region with generally thicker and more compact ice, the ice cover behaves more as an anisotropic continuum with oriented leads and pressure ridges interspersed (Wadhams, 1981). This manifests in observations of oriented failure zones across all spatial scales (Rampal et al., 2008; Stern & Lindsay, 2009; Weiss, 2003). These relatively long and narrow failure zones are usually associated with high ice deformation rates and often referred to as linear kinematic features (LKFs) based on analyses of synthetic aperture radar (SAR) satellite images (Kwok, 2003).

Large‐scale sea ice models of varying complexity are used to simulate the evolution of sea ice. The thickness distribution sea ice models with multiple subgrid‐scale ice thickness categories, based on Thorndike et al. (1975) (also see Hibler, 1980), have wide use (e.g., Hunke & Lipscomb, 2008; Zhang & Rothrock, 2003) because they represent well sea ice of various thicknesses and simulate explicitly the ice ridging process linked closely to ice motion and deformation. These and other sea ice models often treat the sea ice cover as a two‐dimensional continuum obeying a certain constitutive law, or rheology. Ice rheology describes the relationship among ice internal stress, deformation, and mechanical strength and is a key component of the momentum balance that governs the dynamic properties of sea ice.

Perhaps the most widely used rheology in large‐scale sea ice models so far is the isotropic viscous‐plastic (VP) rheology (Hibler, 1979) and its numerical approximation, the elastic‐viscous‐plastic (EVP) method (Hunke & Dukowicz, 1997). This is because of their relative ease in numerical implementation and general success in simulating ice motion and thickness. However, the isotropic VP rheology or EVP method are unable to adequately simulate narrow failure zones of large ice deformation rates when employed in relatively coarse‐resolution (coaser than ∼10 km) sea ice models. This is reflected in the general underestimation of the number and magnitude of narrow LKFs (e.g., Kwok et al., 2008), an indication of their deficiency in representing the anisotropic properties of sea ice in coarse‐resolution models.

As computers become more powerful, there is growing interest in high‐resolution modeling and prediction of sea ice evolution. An increasing number of sea ice models of high horizontal resolution (defined here as < 10 km) have been incorporated into climate or operational forecast models (e.g., Madsen et al., 2015; Posey et al., 2015; Roberts et al., 2015). In particular, the US Navy’s Global Ocean Forecat System (GOFS 3.1) has ∼4‐km horizontal resolution for the Arctic Basin and uses the EVP method to approximate the isotropic VP rheology (Posey et al., 2015).

As model resolutions become ever higher, researchers have studied whether the isotropic VP rheology, developed based on continuum mechanics, is applicable to models of high horizontal resolution. For example, McNutt and Overland (2003) report that sea ice behavior changes from the multifloe scale (defined as 2–10 km) to the aggregate scale (10–75 km). Coon et al. (2007) show that aggregating sea ice as a continuous isotropic material at resolutions higher than ∼10 km cannot be justified, thus casting doubt on the applicability of the isotropic VP rheology in high‐resolution models. Recently, however, an increasing number of studies report that sea ice models with an isotropic VP rheology are able to simulate major LKFs similar to those observed by satellites as model resolutions become sufficiently high (e.g., Hutter et al., 2018; Losch et al., 2014; Spreen et al., 2017; K. Wang & Wang, 2009; Q. Wang et al., 2016). In particular, Hutter et al. (2018), through a scaling analysis, show that sea ice models with an isotropic VP rheology and small horizontal grid spacing (1 km) can resolve sea ice leads and deformation rates localized along LKFs. Thus these recent studies appear to establish the applicability of the VP rheology for sea ice modeling over multiple spatial scales, from the aggregate to multifloe, and possibly beyond.

Given the applicability of an isotropic VP rheology across various scales and its use in high‐resolution sea ice models afforded by increasing computer capabilities, there is a growing interest in investigating sea ice properties in models of varying high resolutions. Are key sea ice properties, such as thickness, concentration, and velocity as well as oriented failure zones (LKFs) sensitive to model resolutions within the multifloe scale? Are these sea ice properties sensitive to parameterizations in models of increasingly high resolutions? These questions have not yet been addressed fully. For example, the mechanical ice strength is an important parameter of the VP rheology that governs when ice fails and enters a state of plastic flow. Thus, it has an important role in determining the speed of ice drift and rate of ice deformation (Hibler, 1979; Rothrock, 1975; Steele et al., 1997). Some studies report that the mechanical ice strength is dependent on model resolution (e.g., Bouchat & Tremblay, 2014; Hutter, 2015; Overland & Pease, 1988; Williams & Tremblay, 2018). And while Bouchat and Tremblay (2014) and Williams and Tremblay (2018) suggest a higher ice strength value for higher model resolution, Overland and Pease (1988) and Hutter (2015) suggest the opposite. These contrasting reports indicate a necessity for further investigation.

This study aims to investigate sea ice properties in models of increasingly high resolutions. By investigating sea ice properties, this study also aims to examine whether and to what degree sea ice model parameters are resolution dependent in models of varying high resolutions within the multifloe scale. Note that mechanical ice strength is just one of many parameters used to model sea ice; others include a range of parameters that control the ridging process in thickness distribution sea ice models (Flato & Hibler, 1995; Hibler, 1980). Instead of assessing the effect of individual parameters one by one, the focus is on what happens if parameters commonly used in coarse‐resolution sea ice models are used similarly in high‐resolution models. How do sea ice properties (e.g., thickness, concentration, velocity, and LKFs) behave in models with varying high resolutions but the same model parameters and atmospheric forcing? This question addresses the relationship between sea ice properties and model resolutions and whether model parameterizations must be adjusted constantly when using increasingly higher resolutions. This will help to guide our development of high‐resolution sea ice models and therefore enhance our ability to predict sea ice evolution. To this end, numerical experiments were designed and conducted using the pan‐arctic High‐resolution Ice‐Ocean Modeling and Assimilation System (HIOMAS), which is constructed with high horizontal resolutions ranging from 6 to 2 km.

## 2 Model Description and Numerical Experiments

HIOMAS was adapted from the Pan‐arctic Ice‐Ocean Modeling and Assimilation System (PIOMAS). It consists of a thickness and enthalpy distribution sea ice model (Zhang & Rothrock, 2003) coupled with the Parallel Ocean Program ocean model (Smith et al., 1992). The sea ice model has eight subgrid‐scale ice thickness categories centered at 0, 0.38, 1.30, 3.07, 5.97, 10.24, 16.02, and 23.4 m and upper‐bounded by 0.1, 0.66, 1.93, 4.2, 7.74, 12.74, 19.31, and 27.51 m, respectively. The first category represents open water. Ice motion in HIOMAS is solved following Zhang and Hibler (1997) based on a momentum equation that consists of a VP rheology with a teardrop plastic yield curve (Zhang & Rothrock, 2005). Ice ridging is simulated following the ridging parameterization of Thorndike et al. (1975) and Hibler (1980). Ice growth or decay is calculated based on the three‐layer thermodynamics of Winton (2000). HIOMAS is capable of assimilating satellite ice concentration data following Lindsay and Zhang (2006). However, in this study satellite ice concentration is not assimilated.

**Figure 1:**
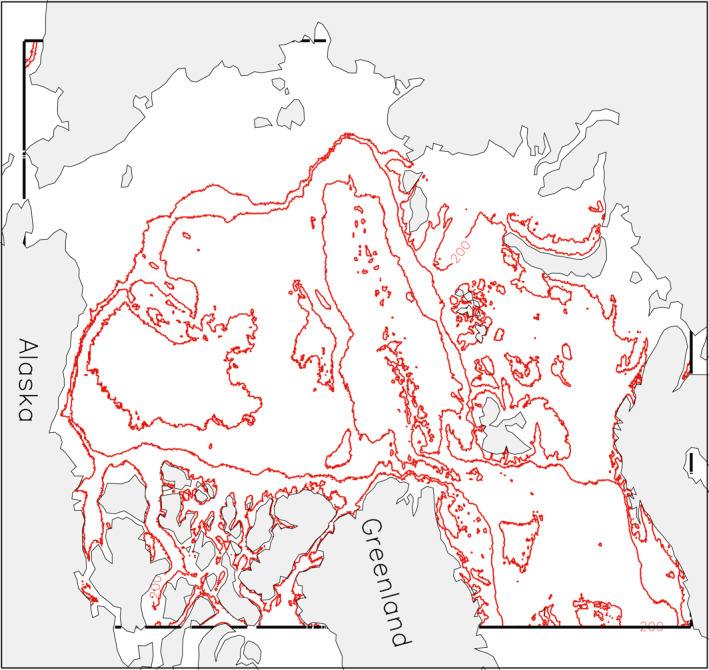
Model domain of the 6‐km resolution HIOMAS enclosed by thick black lines. The 4‐ and 2‐km resolution HIOMAS versions have similar model domains. The red lines represent isobaths of 200, 1,000, and 3600 m. HIOMAS, High‐resolution Ice‐Ocean Modeling and Assimilation System.

To assess whether sea ice properties in high‐resolution models are sensitive to changes in model resolution, three versions of HIOMAS (including both sea ice and ocean model components) were constructed with three uniform horizontal resolutions of 6, 4, and 2 km (i.e., *dx* = *dy* = 6, 4, and 2 km) for the whole Arctic Ocean (see Figure 1 for model domain). Thus, these three versions vary from being eddy‐permitting at 6 km resolution to eddy‐resolving at 2 km resolution (e.g., Nurser & Bacon, 2014). They are designed to have identical sea ice dynamic and thermodynamic parameters, including ice strength and ridging parameters that are used in PIOMAS, which has coarser resolution. For example, ice strength parameterization is based on Hibler (1979) with the strength parameter P* set to 27,500 N m^–1^ following Hibler and Walsh (1982); the parameters governing the ridging process, such as the frictional dissipation coefficient, the ridge participation constant, and shear ridging parameter, are listed in Table 1 (also see Table 3 in Flato & Hibler, 1995 for the standard case). Also listed in Table 1 are the ice strength parameter and ice, snow, and open water albedo values used in the three HIOMAS versions. To ensure that the sea ice model component is subject to similar oceanic forcing, the three HIOMAS versions all have 40 ocean levels, with identical ocean parameters such as viscosity and diffusivity, although they differ in their ability to resolve eddies. They are all one‐way nested to a global coupled ice‐ocean model (Zhang, 2005) and driven by the same daily NCEP/NCAR reanalysis atmospheric forcing including 10‐m surface winds, 2‐m surface air temperature, and longwave and shortwave radiation with a spatial resolution of ∼2.5° × 2.5°, which are interpolated onto the model grids of different resolutions. Forced by the NCEP/NCAR reanalysis, they are all integrated over the period 2011–2018, initialized with a constant 2‐m ice thickness in the areas of surface air temperature at or below 0°C on the first day of 2011, zero ice and ocean velocity, and ocean temperature and salinity climatology (Levitus, 1982).

**Table 1:** Some Sea Ice Parameters Used in the Different HIOMAS Model Versions

Parameters	Value
Sea ice strength parameter P*	27,500 N m^–1^
Frictional dissipation coefficient in ridging	17
Ridge participation constant	0.15
Shear ridging parameter	0.5

Abbreviation: HIOMAS, High‐resolution Ice‐Ocean Modeling and Assimilation System.

**Figure 2:**
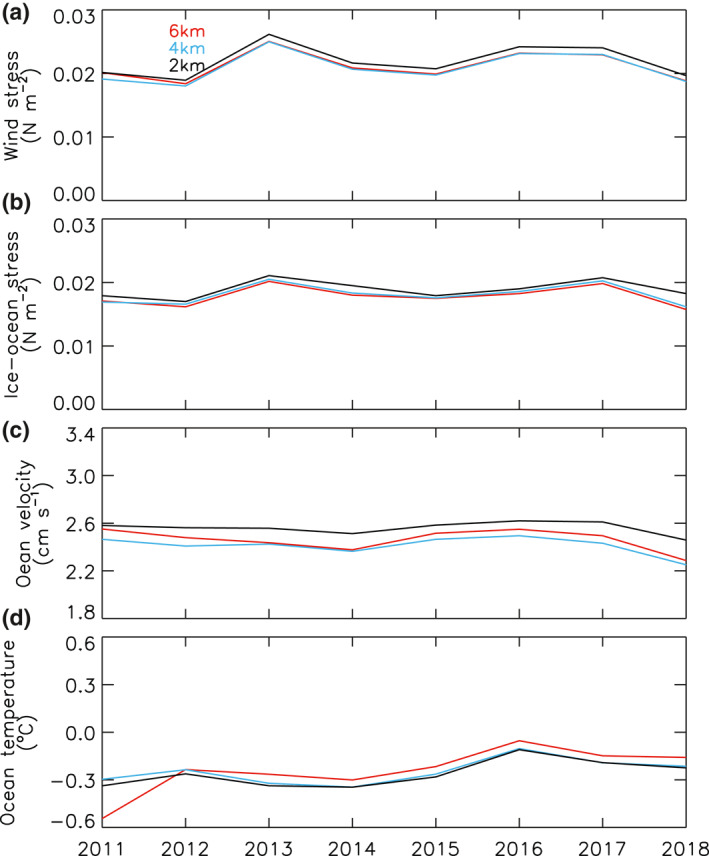
Simulated annual mean wind stress on the surface of ice and open water (a), stress at the ice‐ocean interface (b), and ocean velocity (c) and temperature (d) in the upper 30 m, averaged over the Arctic Ocean defined as the whole model domain (see Figure 1), from the three versions of HIOMAS for the period 2011–2018. HIOMAS, High‐resolution Ice‐Ocean Modeling and Assimilation System.

To make sure that the three HIOMAS versions, which are subject to the same atmospheric forcing interpolated to their individual model grids, are also subject to similar oceanic forcing, ocean velocity and temperature in the upper ocean (30 m) and the stress at the ice‐ocean interface averaged over the Arctic Ocean are examined (Figure 2). The ocean velocity in the upper 30 m is lower in the first year of integration (2011) than most of the other years, due partially to the initial conditions of zero ocean velocity (Figure 2a). It starts to increase in 2013 and peaks in 2016 before decreasing toward 2018. Thus, the upper ocean velocity is more or less stabilized over the period 2012–2018, without continuous decrease or increase. The ocean velocity from the 2‐km resolution HIOMAS is generally higher than the other two versions, likely due to the fact that it is eddy resolving. However, the relative differences among these three versions are small, within 5%.

Stress at the ice‐ocean interface is associated with the upper ocean velocity (Hibler, 1979). It is not surprising that the simulated stress under ice is also in a generally steady state. Like the upper ocean velocity, the stress has slightly higher values with the 2‐km resolution HIOMAS (Figure 2b). The relative differences among these three versions are also within 5%. In addition, the differences in ocean temperature in the upper 30 m among the three HIOMAS versions are small as well (Figure 2c). All these indicate that the sea ice processes in these models of different high resolutions are driven by similar dynamic thermodynamic oceanic forcing.

## 3 Results

**Figure 3:**
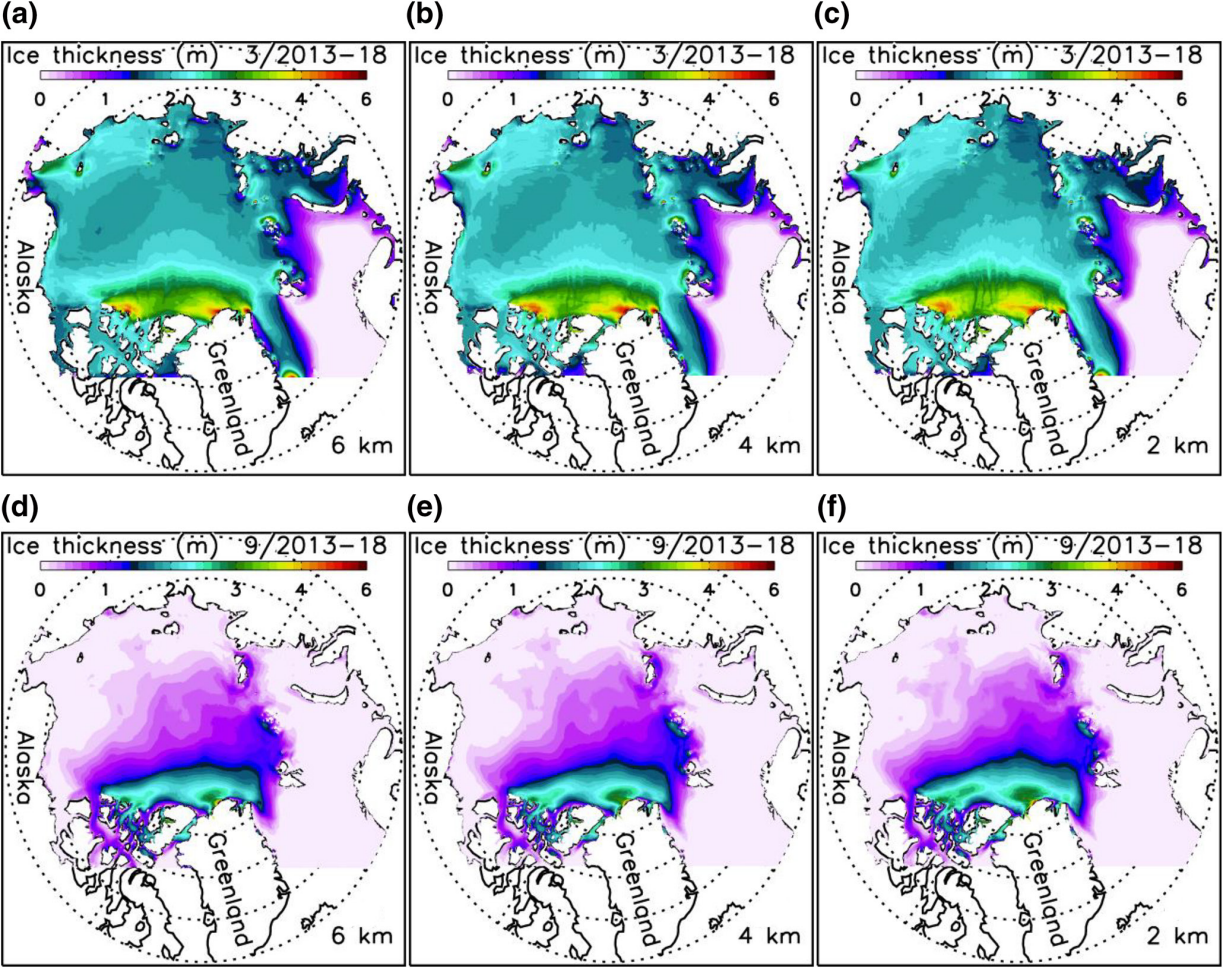
Simulated 2013–2018 March (a, b, and c) and September (d, e, and f) mean sea ice thickness from three versions of HIOMAS with horizontal resolutions of 6, 4, and 2 km. HIOMAS, High‐resolution Ice‐Ocean Modeling and Assimilation System.

HIOMAS captures the basic spatial pattern of sea ice thickness in winter and summer (Figure 3). The simulated thick ice generally stays in the areas near the Canadian Archipelago and northern Greenland and thinner ice elsewhere, including the Beaufort Sea, where ice is mostly under 2.5 m thick in March (Figures 3a–3c) and largely melts away in September (Figures 3d–3f). Visually, there are some noticeable differences in ice thickness near the Canadian Archipelago in March among the three HIOMAS versions. However, the differences appear to be relatively small in limited areas. On average, all three versions show similar spatial patterns and temporal variations of ice thickness fields.

**Figure 4:**
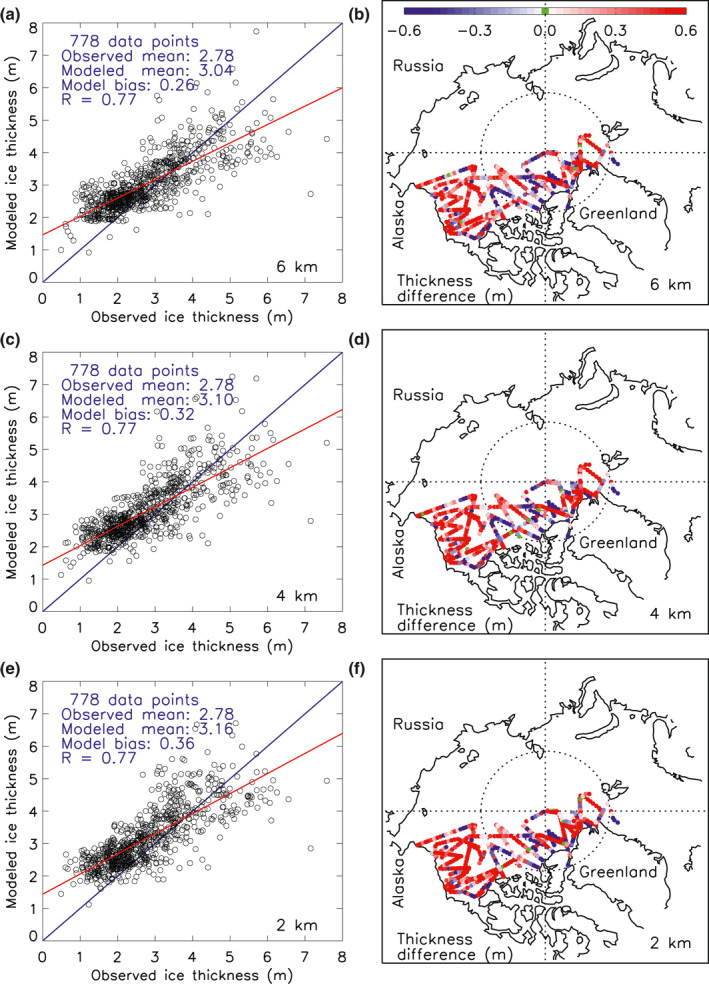
(a), (c), and (e) Simulated sea ice thickness (m) from three versions of HIOMAS and thickness data from the NASA IceBridge program (Kurtz et al., 2013) collected during March and/or April of 2013–2015 and available from the Unified Sea Ice Thickness Climate Data Record (Lindsay, 2010). The blue line indicates equality and the red line represents the best fit to the observations. The number of total observation points, observed and modeled mean values, model bias (mean model–observation difference), and model–observation correlation (R) are listed. (b), (d), and (f) Point‐by‐point differences in ice thickness (m) between model results and observations at the locations of the observations. Red dots indicate that the modeled ice is thicker than the observed and blue dots thinner. HIOMAS, High‐resolution Ice‐Ocean Modeling and Assimilation System.

The similarity in mean ice thickness fields is also reflected in a comparison with NASA IceBridge sea ice thickness observations collected during 2013–2015 (Figure 4). The three versions all tend to underestimate thick ice and overestimate thinner ice (Figure 4, left panels). This is in part because of model bias and in part because models often create fields that are likely smoother than point or line measurements and therefore unable to reproduce some of the thick or thin ice in observations, even with HIOMAS horizontal resolutions of 6–2 km. All versions overestimate ice thickness in the Beaufort Sea (Figure 4, more red dots than blue dots), but the overestimation is mostly below half a meter (Figure 4, right panels). Near the Canadian Archipelago, northern Greenland, and Fram Strait, positive and negative thickness differences are nearly evenly distributed, indicating a relatively low mean model bias in those areas. Overall, all model versions behave alike when compared to NASA IceBridge ice thickness observations, with a small variation of mean model bias (0.26–0.36 m) and the same model‐observation correlation (0.77) and root‐mean‐square error (0.73 m, not listed) (Figure 4, left panels). Increasing model resolution from 6 to 2 km does not significantly improve the overall model performance compared to IceBridge observations.

**Figure 5:**
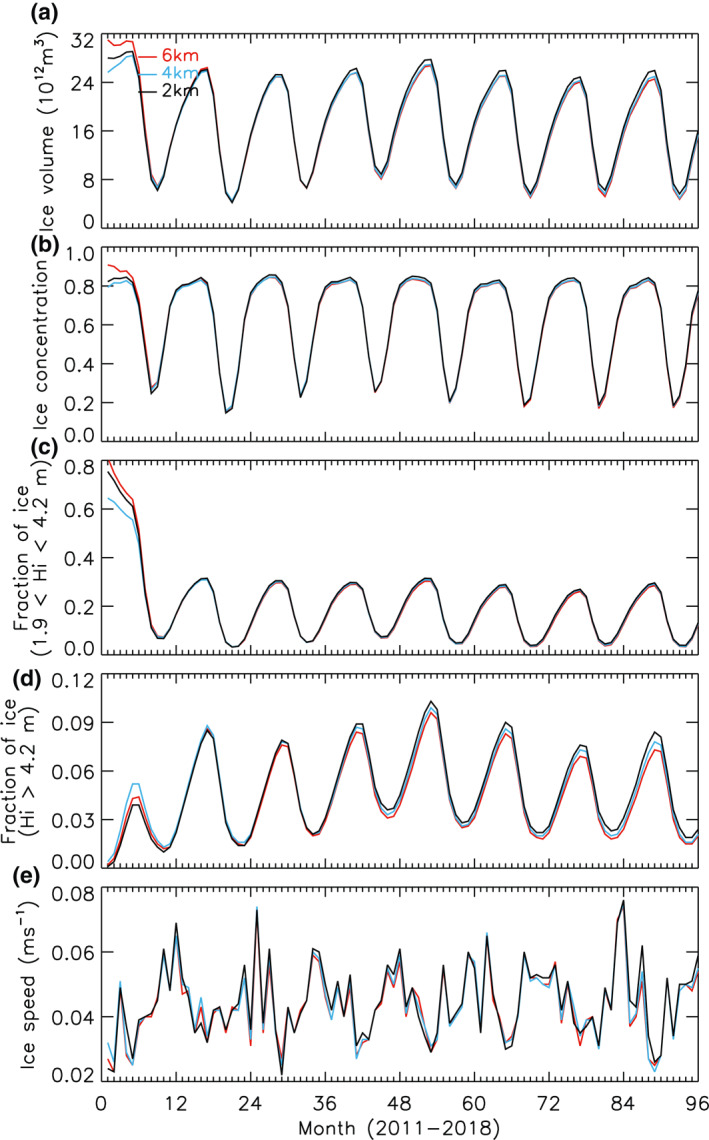
(a) Monthly mean total sea ice volume in the Arctic Ocean defined as the whole model domain (see Figure 1) and (b) ice concentration, (c) fraction of ice thicker than 1.9 m and thinner than 4.2 m (category 4), (d) fraction of ice thicker than 4.2 m (categories 5–8), and (e) ice speed averaged over the Arctic Ocean, simulated by the three versions of HIOMAS for the period 2011–2018. HIOMAS, High‐resolution Ice‐Ocean Modeling and Assimilation System.

The magnitude and seasonal evolution of the total sea ice volume over the period 2012–2018 from the three HIOMAS versions are almost identical (Figure 5a) after the first integration year 2011. The ice volume from the 2 km resolution model appears to be slightly higher than the other two models, suggesting a tendency for ice volume to increase with increasing high model resolutions. However, the increase is small and the ice volume difference among the three model runs over 2012–2018 is within 2%. This is not surprising, given that the three different model runs create similar spatiotemporal variability of ice thickness fields over the Arctic Ocean (Figures 3 and 4). The magnitude and seasonal evolution of mean ice concentration over the Arctic Ocean are also almost identical over 2012–2018 (Figure 5b), because of similar spatiotemporal variability of ice concentration fields (not shown). In addition, the magnitude and seasonal evolution of the fraction of ice thicker than 1.9 m and thinner than 4.2 m (category 4; Figure 5c), the fraction of ice thicker than 4.2 m (categories 5–8; Figure 5d), and the fraction of ice thinner than 1.9 m (categories 1–3; not shown), averaged over the Arctic Ocean are all nearly identical for the three HIOMAS runs after 2011. In association with a slightly higher ice volume (Figure 5a), the 2 km resolution model creates a slightly higher fraction of ice thicker than 4.2 m than the other two models (Figure 5d). However, the difference in the fraction of ice thicker than 4.2 m among the three models over 2012–2018 is small, within 4%. Thus, the spatiotemporal variability of the 8‐category subgrid ice thickness distribution is about the same among the three versions.

The insignificant change in the 8‐category ice thickness distribution among the three versions is an indication that the ridging parameters used in HIOMAS, following Flato and Hibler (1995), are not a function of model resolution within the multifloe scale. In other words, changing the model resolution does not significantly change the behavior of the subgrid scale ice thickness distribution. This is because ice thickness distribution is strongly influenced by the ridging process that transfers thin ice into thick ice categories through mechanical thickness redistribution (Hibler, 1980; Thorndike et al., 1975). It is also an indication that within the multifloe scale the VP rheology and the mechanical ice strength parameterization behave similarly in all resolutions because the ice ridging process is driven by ice motion and deformation, which are largely controlled by the VP rheology and ice strength in a thickness distribution sea ice model (Hibler, 1980; Thorndike et al., 1975). In fact, the similar spatiotemporal variability of mean ice thickness and concentration fields and the nearly identical magnitude and seasonal evolution of the total ice volume and mean ice concentration and fractions of various ice thickness categories suggest that the large‐scale sea ice properties of high‐resolution sea ice models, with the same dynamic and thermodynamic parameters and under similar atmospheric and oceanic forcing, are insensitive to varying model resolutions.

**Figure 6:**
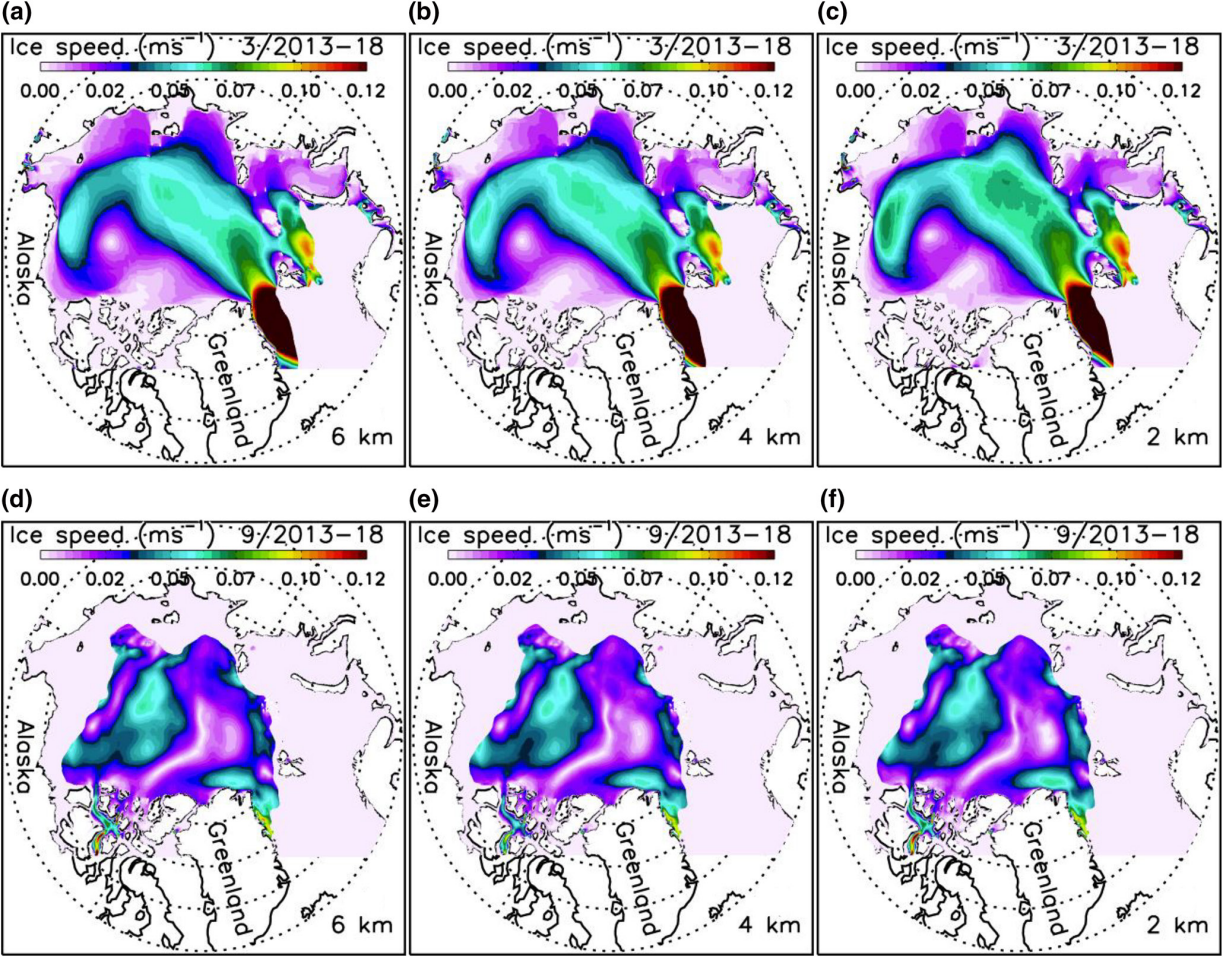
Simulated 2013–2018 March (a, b, and c) and September (d, e, and f) mean sea ice speed from three versions of HIOMAS. HIOMAS, High‐resolution Ice‐Ocean Modeling and Assimilation System.

Given that the VP rheology and the ice strength parameter are key in determining ice motion (Hibler, 1979), their insensitivity to different high model resolutions is also reflected in the simulated mean fields of ice speed for March and September 2013–2018 (Figure 6). The March mean ice motion pattern has typical winter conditions with robust Beaufort Gyre anticyclonic circulation, transpolar drift, and East Greenland Current (Figures 6a–6c). In September, ice motion is generally weaker than in March (Figures 6d–6f). There are some differences in ice speed among the three HIOMAS versions, which may be linked to their difference in ability to resolve eddies. However, the differences are limited and the ice speed fields from the three versions largely resemble each other for both winter and summer. This is why the magnitude and seasonal evolution of the ice speed averaged over the Arctic Ocean do not differ significantly among the three versions (Figure 5e). The similar spatiotemporal variability of ice motion contributes to the similar spatiotemporal variability of ice thickness and the nearly identical magnitude and seasonal evolution of ice volume, ice concentration, and area fractions of various ice thickness categories.

**Figure 7:**
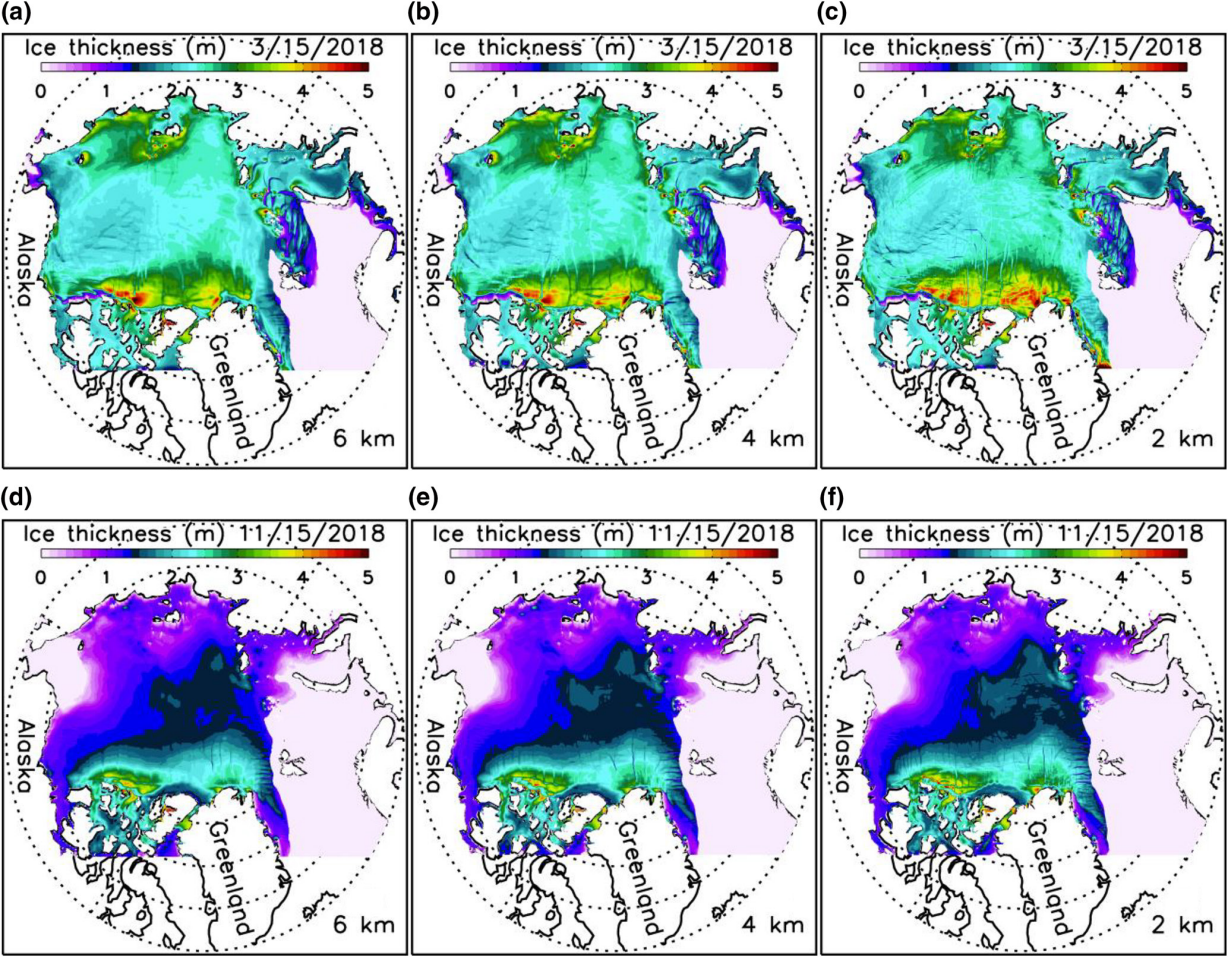
Simulated sea ice thickness for March 15 (a, b, and c) and November 15 (d, e, and f), 2018 from three versions of HIOMAS. HIOMAS, High‐resolution Ice‐Ocean Modeling and Assimilation System.

**Figure 8:**
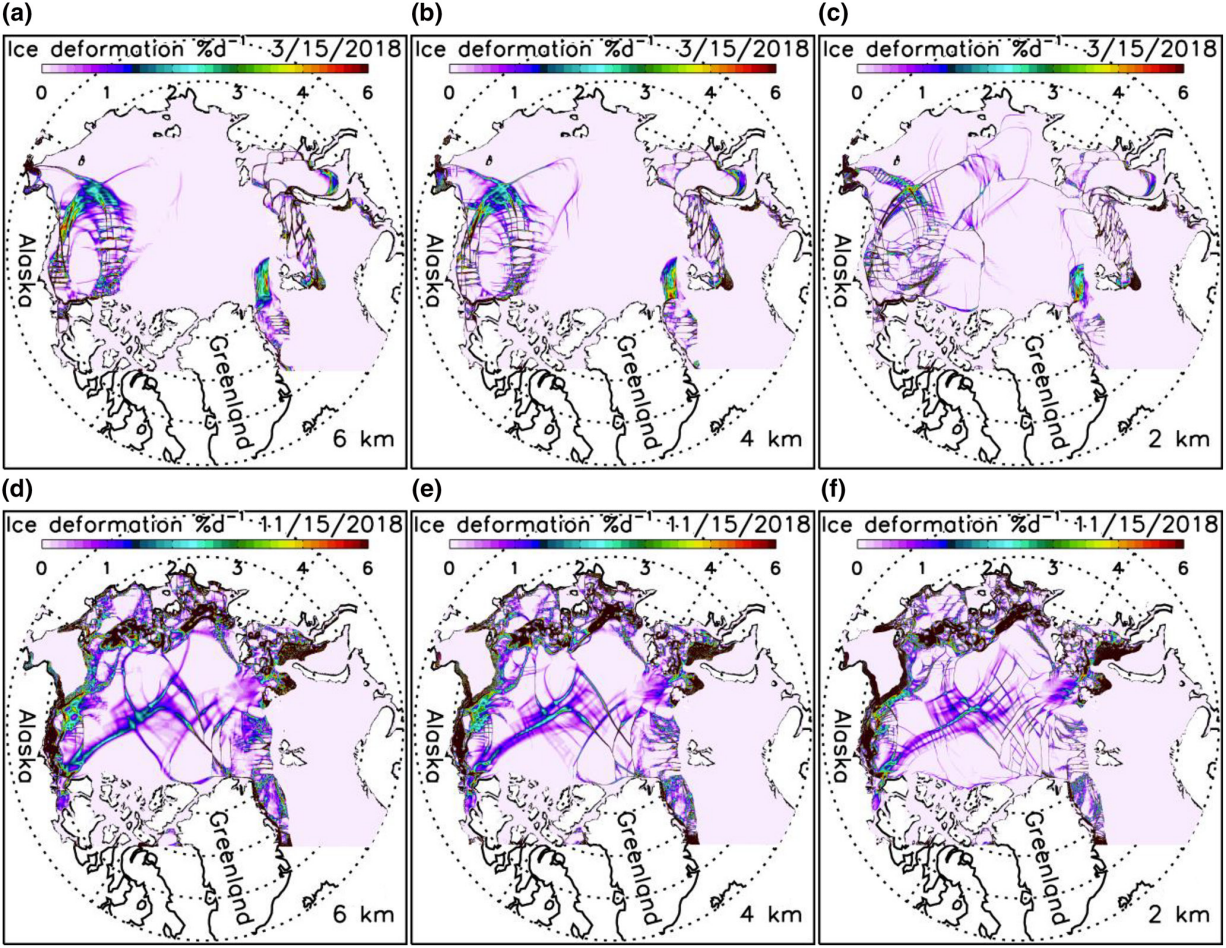
Simulated sea ice deformation rate for March 15 (a, b, and c) and November 15 (d, e, and f), 2018 from three versions of HIOMAS. HIOMAS, High‐resolution Ice‐Ocean Modeling and Assimilation System.

A careful examination of daily winter ice thickness fields indicates that the three high‐resolution models are able to simulate some major oriented narrow sea ice leads or failure zones (Figures 7a–7c). This is also reflected in the simulated fields of winter ice deformation rate ($\sqrt{{\left({\dot{e}}_{11}+{\dot{e}}_{22}\right)}^{2}+\left[{\left({\dot{e}}_{11}-{\dot{e}}_{22}\right)}^{2}+4{\dot{e}}_{12}^{2}\right]}$, where ${\dot{e}}_{ij}$ is strain rate tensor) localized along LKFs (Figures 8a–8c). These deformation fields show a general spatial pattern of LKFs qualitatively in agreement with that seen in SAR satellite images (Kwok, 2003), as also reported by Hutter et al. (2018) via a scaling analysis. However, the simulated spatial distributions of sea ice leads and deformation rates are different among the three model versions. They differ in the number, location, shape, width, length, and orientation of leads or LKFs. This suggests that, although they create similar spatial patterns and temporal variations of ice thickness fields, they differ from each other in representing anisotropic properties of sea ice in winter. In areas of thin ice, few narrow failure zones are simulated by these model versions (Figures 7d–7f) because of weaker mechanical ice strength leading to generally large ice deformation (Figures 8d–8f).

**Figure 9:**
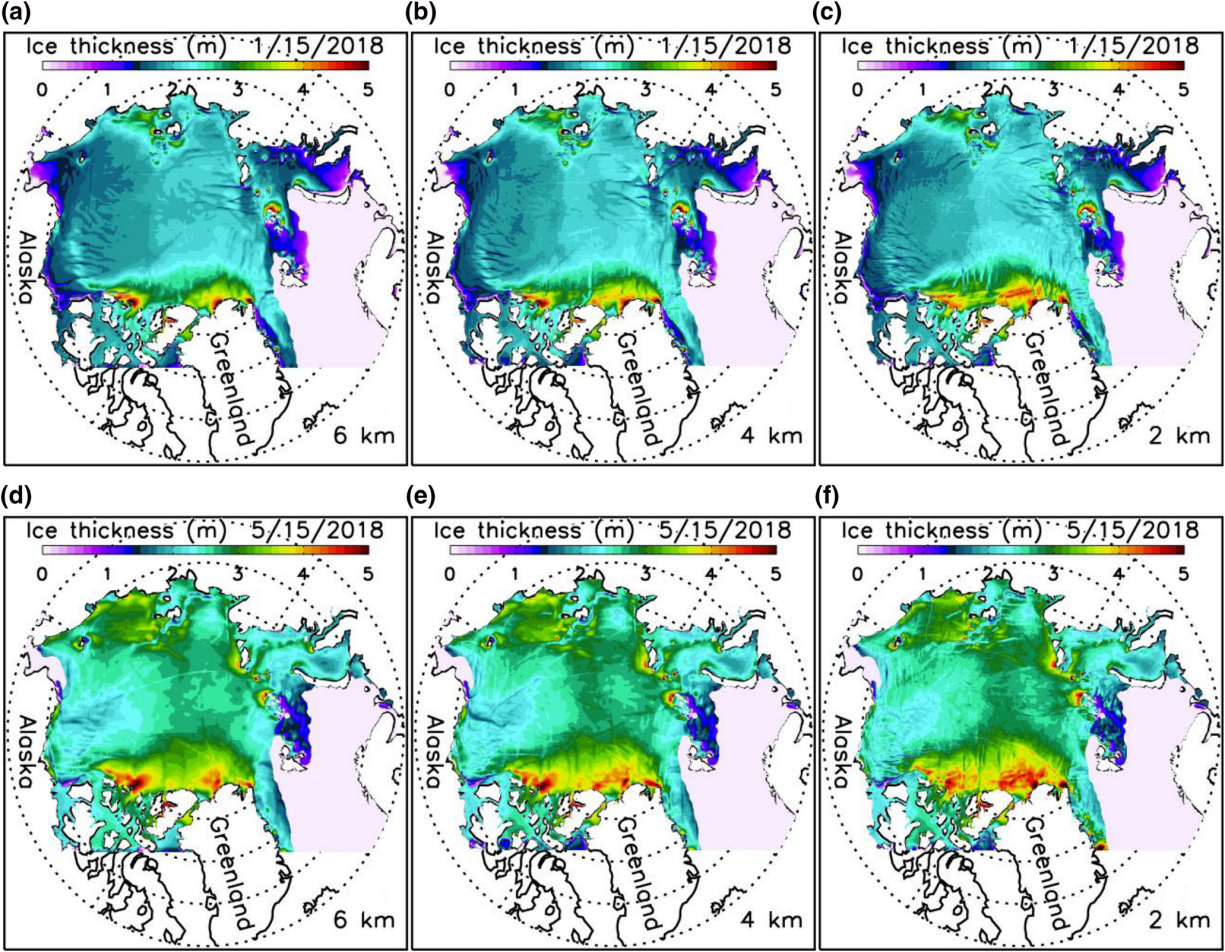
Simulated sea ice thickness for January 15 (a, b, and c) and May 15 (d, e, and f), 2018 from three versions of HIOMAS. HIOMAS, High‐resolution Ice‐Ocean Modeling and Assimilation System.

**Figure 10:**
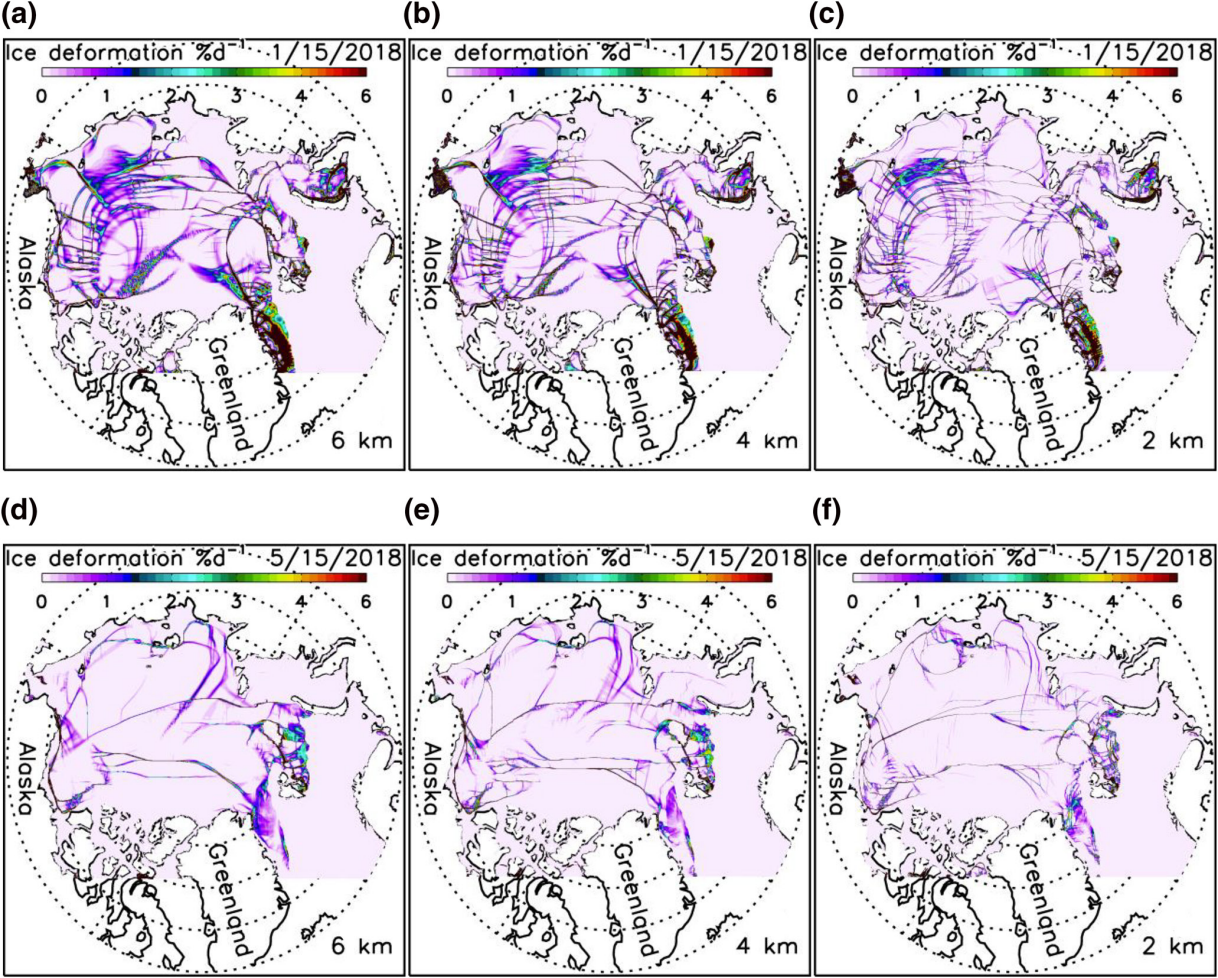
Simulated sea ice deformation rate for January 15 (a, b, and c) and May 15 (d, e, and f), 2018 from three versions of HIOMAS. HIOMAS, High‐resolution Ice‐Ocean Modeling and Assimilation System.

Although a quantitative scaling analysis like that of Hutter et al. (2018) is beyond the scope of this study, it is apparent visually from both ice thickness and ice deformation fields that the 2‐km resolution HIOMAS tends to create a greater number of oriented failures zones in winter that are often narrower than the other two versions (Figures 7a–7c and 8a–8c). This is also illustrated in additional ice thickness and deformation fields for winter and spring conditions (Figures 9 and 10). These results confirm previous studies showing that high‐resolution sea ice models with an isotropic VP rheology capture generally the anisotropic behavior of sea ice (e.g., Hutter et al., 2018; Losch et al., 2014; Spreen et al., 2017; K. Wang & Wang, 2009; Q. Wang et al., 2016). They further indicate that sea ice models with increasingly high resolutions are likely to better resolve the narrow leads or failure zones, which is important to maritime operations.

## 4 Discussion and Conclusions

Three versions of HIOMAS with an ice thickness distribution, an isotropic VP rheology, and three different high resolutions within the multifloe scale (2–10 km; McNutt & Overland, 2003) produce nearly invariant mean sea ice properties. All three model versions create generally similar spatial patterns and temporal variations of mean ice thickness, concentration, and motion, three key ice state variables, and nearly identical magnitude and seasonal evolution of the total ice volume and mean ice concentration, ice speed, and fractions of ice of various thickness categories. In particular, they all compare reasonably well with the NASA IceBridge observations of ice thickness, with slightly different mean model biases and the same model‐observation correlation and root‐mean‐square error. This means that the large‐scale sea ice properties of HIOMAS are insensitive to changes in resolution within the multifloe scale under similar atmospheric and oceanic forcing. It is unnecessary to constantly adjust model parameters when models use increasingly higher resolutions.

Modeling ice dynamic processes, such as ice motion, deformation, and ridging, is thought to be influenced by changes in model horizontal resolution (e.g., Bouchat & Tremblay, 2014; Hutter, 2015; Overland & Pease, 1988; Williams & Tremblay, 2018). Yet results from this study show that the ice dynamics parameterizations that control ice dynamic processes, such as those for the VP rheology, mechanical ice strength, and ice ridging, are largely independent of model resolution likely because they do not use any grid spacing information. This applies with models of high horizontal resolutions (<10 km) within the multifloe scale. It also applies with models of coarser resolutions (>10 km) outside of the multifloe scale. This is reflected in an additional numerical experiment conducted using a fourth HIOMAS version with 12 km resolution. From 2 to 12 km, the range of spatial resolution among the four model versions has been expanded by a factor of 6. Results from the 12 km resolution model, including sea ice motion, concentration, thickness, and volume, are close to those from the other three cases (not shown). In fact, the VP rheology and the parameterizations of ice strength and ridging in all four HIOMAS versions are the same as PIOMAS, a model with coarser horizontal resolutions (∼15–∼70 km), which is within the aggregate scale (10–75 km) defined by McNutt and Overland (2003). Thus, the isotropic VP rheology and the parameterizations of ice strength and ridging are applicable for both multifloe and aggregate scales in simulating large‐scale or climate‐scale sea ice properties. In other words, the VP rheology and the parameterizations used at coarse resolution may be used at high resolution because the three high‐resolution models give plausible results. In particular, for those multiscale modern sea ice models with varying spatial resolutions from the multifloe scale to the aggregate scale, there is probably no need to implement spatially varying VP rheology and ice strength and ridging parameters.

Although increasing model resolution changes the mean state of sea ice little, it improves, on a daily time scale, the simulation of relatively long and narrow sea ice leads or failure zones that are ubiquitous in the Arctic sea ice cover in winter and spring. To a varying degree, the three HIOMAS versions with horizontal resolutions of 6, 4, and 2 km all create some of the major leads and LKFs with characteristics similar to SAR observations (Kwok, 2003). While a detailed scaling analysis is not intended in this study, the 2‐km resolution HIOMAS better resolves leads than the other two versions, creating generally a greater number of failures zones that are often narrower. This is reflected in daily ice thickness and ice deformation fields throughout winter and spring. The ability to resolve more leads or failure zones may stimulate ice growth under freezing conditions, contributing to slight increases in the fraction of thick ice and hence ice volume (Figure 5). Sea ice prediction from higher‐resolution models is likely to be of greater use for Arctic operators such as sailors, oil and gas workers, and fishers who are interested in lead distribution information.

The isotropic VP rheology and its associated ice strength parameterization assume that the ice cover is a two‐dimensional continuum. In this study, the realistic simulation of sea ice thickness in agreement with the NASA IceBridge observations and ice deformation localized along failure zones in qualitative agreement with SAR observations demonstrate the usefulness of the VP rheology in representing anisotropic properties of sea ice in models with resolutions up to 2 km. Hutter et al. (2018) report its usefulness in creating realistic LKFs in a model with 1‐km resolution, although no ice thickness results were presented and compared with observations. Would the VP rheology fail in models with resolution even higher than 1 km (tens to hundreds of meters) because of a potential breakdown of the assumed continuum mechanics as the model resolution becomes smaller than the size of many individual ice floes? The answer appears to be no, based on recent studies such as Heorton et al. (2018) and Ringeisen et al. (2019) who were able to simulate even narrower LKFs using idealized models with resolution significantly higher than 1 km. However, how would key sea ice properties, such as ice motion, deformation, thickness, and volume, behave in realistic models at such high resolution (<1 km)? Also, would realistic models at such high resolution need to adjust model parameterizations when there is a change in model resolution? These questions warrant further studies.

The numerical experiments presented here only involve sea ice‐ocean models that are driven by fixed atmospheric forcing interpolated to the individual model grids. They do not involve fully coupled models with an atmospheric model component. Thus, it is not clear whether and to what degree sea ice and ocean model components at an increasingly high resolution may impact the atmosphere model component through air–ice–sea interactions, which may, in turn, affect sea ice motion, concentration, and thickness. Can a fully coupled air‐ice‐sea model better resolve ice leads at a high resolution? Do we need to adjust sea ice or other model parameterizations constantly with increasing resolution of a fully coupled model? These questions also warrant further studies.

## Data Availability

Model results of ice thickness and deformation are available in https://pscfiles.apl.uw.edu/zhang/HIOMAS/.

## References

[jgrc24319-bib-0001] Bouchat, A. , & Tremblay, B. (2014). Energy dissipation in viscous‐plastic sea‐ice models. Journal of Geophysical Research: Oceans, 119(2), 976–994. 10.1002/2013JC009436

[jgrc24319-bib-0002] Coon, M. D. , Kwok, R. , Levy, G. , Pruis, M. , Schreyer, H. , & Sulsky, D. (2007). Arctic ice dynamics joint experiment (AIDJEX) assumptions revisited and found inadequate. Journal of Geophysical Research, 112, C11S90. 10.1029/2005jc003393

[jgrc24319-bib-0003] Flato, G. M. , & Hibler, W. D., III (1995). Ridging and strength in modeling the thickness distribution of Arctic sea ice. Journal of Geophysical Research, 100, 18611–18626.

[jgrc24319-bib-0004] Heorton, H. D. B. S. , Feltham, D. L. , & Tsamados, M. (2018). Stress and deformation characteristics of sea ice in a high‐resolution, anisotropic sea ice model. Philosophical Transactions of the Royal Society A, 376, 20170349. 10.1098/rsta.2017.0349 PMC6107622 30126920

[jgrc24319-bib-0005] Hibler, W. D., III (1979). A dynamic thermodynamic sea ice model. Journal of Physical Oceanography, 9, 815–846.

[jgrc24319-bib-0006] Hibler, W. D., III (1980). Modeling a variable thickness sea ice cover. Monthly Weather Review, 108, 1943–1973.

[jgrc24319-bib-0007] Hibler, W. D., III , & Walsh, J. E. (1982). On modelling seasonal and interannual fluctuation of Arctic sea ice. Journal of Physical Oceanography, 12, 1514–1523.

[jgrc24319-bib-0008] Hunke, E. C. , & Dukowicz, J. K. (1997). An elastic‐viscous‐plastic model for sea ice dynamics. Journal of Physical Oceanography, 27, 1849–1867.

[jgrc24319-bib-0009] Hunke, E. C. , & Lipscomb, W. H. (2008). CICE: The Los Alamos sea ice model (Documentation and software user's manual version 4.0, Tech. Rep. LA‐CC‐06‐012, T‐3 Fluid Dyn. Group). Los Alamos, NM: Los Alamos Natl. Lab.

[jgrc24319-bib-0010] Hutter, N. (2015). Viscous plastic sea ice models at very high resolution, Bremen, Germany: (Master thesis): University of Bremen, Alfred Wegener Institute hdl:10013/epic.46129.

[jgrc24319-bib-0011] Hutter, N. , Losch, M. , & Menemenlis, D. (2018). Scaling properties of Arctic sea ice deformation in a high‐resolution viscous plastic sea ice model and in satellite observations. Journal of Geophysical Research: Oceans, 123, 672–687. 10.1002/2017JC013119 29576996 PMC5856068

[jgrc24319-bib-0012] Kurtz, N. T. , Farrell, S. L. , Studinger, M. , Galin, N. , Harbeck, J. P. , Lindsay, R. , et al. (2013). Sea ice thickness, freeboard, and snow depth products from Operation IceBridge airborne data. The Cryosphere, 7, 1035–1056. 10.5194/tc-7-1035-2013

[jgrc24319-bib-0013] Kwok, R. (2003). RGPS Arctic Ocean sea ice deformation from SAR ice motion: Linear kinematic features Winter 1996–1997, Winter 1997–1998, Summer 1998 (p. 80). Pasadena, CA: Rep. JPL D‐21524. Polar Remote Sensing Group, Jet Propulsion Laboratory.

[jgrc24319-bib-0014] Kwok, R. , Hunke, E. C. , Maslowski, W. , Menemenlis, D. , & Zhang, J. (2008). Variability of sea ice simulations assessed with RGPS kinematics. Journal of Geophysical Research, 113, C11012. 10.1029/2008JC004783

[jgrc24319-bib-0015] Levitus, S. (1982). Climatological atlas of the world ocean (NOAA Professional Paper No. 13, p. 173). U.S. Govt. Printing Office.

[jgrc24319-bib-0016] Lindsay, R. W. (2010). Unified sea ice thickness climate data record, Seattle, WA: University of Washington, Polar Science Center, Applied Physics Laboratory. psc.apl.washington.edu/sea_ice_cdr, digital media.

[jgrc24319-bib-0017] Lindsay, R. W. , & Zhang, J. (2006). Assimilation of ice concentration in an ice–ocean model. Journal of Atmospheric and Oceanic Technology, 23, 742–749.

[jgrc24319-bib-0018] Losch, M. , Fuchs, A. , Lemieux, J.‐F. , & Vanselow, A. (2014). A parallel Jacobian‐free Newton–Krylov solver for a coupled sea ice–ocean model. Journal of Computational Physics, 257, 901–911. 10.1016/j.jcp.2013.09.026

[jgrc24319-bib-0019] Madsen, K. S. , Rasmussen, T. A. S. , Ribergaard, M. H. , & Ringgaard, I. M. (2015). High resolution sea ice modelling and validation of the Arctic with focus on south Greenland waters, 2004‐2013. Polarforschung, 85(2), 101–105. 10.2312/polfor.2016.006

[jgrc24319-bib-0020] McNutt, S. L. , & Overland, J. E. (2003). Spatial hierarchy in Arctic sea ice dynamics. Tellus Series A, 55(2), 181–191.

[jgrc24319-bib-0021] Nurser, A. , & Bacon, S. (2014). The Rossby radius in the Arctic Ocean. Ocean Science, 10, 967–975. 10.5194/os-10-967-2014

[jgrc24319-bib-0022] Overland, J. E. , & Pease, C. H. (1988). Modeling ice dynamics of coastal seas. Journal of Geophysical Research, 93(C12), 15619–15637.

[jgrc24319-bib-0023] Posey, P. G. , Metzger, E. J. , Wallcraft, A. J. , Hebert, D. A. , Allard, R. A. , & Smedstad, O. M. , et al. (2015). Improving Arctic sea ice edge forecasts by assimilating high horizontal resolution sea ice concentration data into the US Navy's ice forecast systems. The Cryosphere, 9, 1735–1745. 10.5194/tc-9-1735-2015

[jgrc24319-bib-0024] Rampal, P. , Weiss, J. , Marsan, D. , Lindsay, R. , & Stern, H. (2008). Scaling properties of sea ice deformation from buoy dispersion analysis. Journal of Geophysical Research, 113, C03002. 10.1029/2007JC004143

[jgrc24319-bib-0025] Ringeisen, D. , Losch, M. , Tremblay, L. B. , & Hutter, N. (2019). Simulating intersection angles between conjugatefaults in sea ice with different viscous–plastic rheologies. The Cryosphere, 13, 1167–1186. 10.5194/tc-13-1167-2019

[jgrc24319-bib-0026] Roberts, A. F. , Craig, A. , Maslowski, W. , Osinski, R. , Duvivier, A. , Hughes, M. , et al. (2015). Simulating transient ice‐ocean Ekman transport in the Regional Arctic System Model and Community Earth System Model. Annals of Glaciology, 56(69), 211–228. 10.3189/2015AoG69A760

[jgrc24319-bib-0027] Rothrock, D. A. (1975). The energetics of the plastic deformation of pack ice by ridging. Journal of Geophysical Research, 80, 4514–4519.

[jgrc24319-bib-0028] Rothrock, D. A. , & Thorndike, A. S. (1984). Measuring the sea ice floe size distribution. Journal of Geophysical Research, 89, 6477–6486.

[jgrc24319-bib-0029] Smith, R. D. , Dukowicz, J. K. , & Malone, R. C. (1992). Parallel ocean general circulation modeling. Physica D, 60, 38–61.

[jgrc24319-bib-0030] Spreen, G. , Kwok, R. , Menemenlis, D. , & Nguyen, A. T. (2017). Sea ice deformation in a coupled ocean–sea‐ice model and in satellite remote sensing data. The Cryosphere, 11, 1553–1573. 10.5194/tc-11-1553-2017

[jgrc24319-bib-0031] Steele, M. , Zhang, J. , Rothrock, D. A. , & Stern, H. (1997). The force balance of sea ice in a numerical model of the Arctic Ocean. Journal of Geophysical Research, 102, 21061–21079.

[jgrc24319-bib-0032] Stern, H. L. , & Lindsay, R. W. (2009). Spatial scaling of arctic sea ice deformation. Journal of Geophysical Research, 114, C10017. 10.1029/2009JC005380

[jgrc24319-bib-0033] Thorndike, A. S. , Rothrock, D. A. , Maykut, G. A. , & Colony, R. (1975). The thickness distribution of sea ice. Journal of Geophysical Research, 80, 4501–4513.

[jgrc24319-bib-0034] Wadhams, P. (1981). Sea‐ice topography of the Arctic Ocean in the region 70^o^W to 25^o^E. Philosophical Transactions of the Royal Society of London, 302, 45–85.

[jgrc24319-bib-0035] Wang, Q. , Danilov, S. , Jung, T. , Kaleschke, L. , & Wernecke, A. (2016). Sea ice leads in the Arctic Ocean: Model assessment, interannual variability and trends. Geophysical Research Letters, 43, 7019–7027. 10.1002/2016GL068696

[jgrc24319-bib-0036] Wang, K. , & Wang, C. (2009). Modeling linear kinematic features in pack ice. Journal of Geophysical Research, 114, C12011. 10.1029/2008JC005217

[jgrc24319-bib-0037] Weiss, J. (2003). Scaling of fracture and faulting of ice on earth. Surveys in Geophysics, 24(2), 185–227. 10.1023/A:1023293117309

[jgrc24319-bib-0038] Williams, J. , & Tremblay, L. B. (2018). The dependence of energy dissipation on spatial resolution in a viscous‐plastic sea‐ice model. Ocean Modelling, 130, 40–47.

[jgrc24319-bib-0039] Winton, M. (2000). A reformulated three‐layer sea ice model. Journal of Atmospheric and Oceanic Technology, 17, 525–531.

[jgrc24319-bib-0040] Zhang, J. (2005). Warming of the arctic ice‐ocean system is faster than the global average since the 1960s. Geophysical Research Letters, 32, L19602. 10.1029/2005GL024216

[jgrc24319-bib-0041] Zhang, J. , & Hibler, W. D. (1997). On an efficient numerical method for modeling sea ice dynamics. Journal of Geophysical Research, 102, 8691–8702.

[jgrc24319-bib-0042] Zhang, J. , & Rothrock, D. A. (2003). Modeling global sea ice with a thickness and enthalpy distribution model in generalized curvilinear coordinates. Monthly Weather Review, 131(5), 681–697.

[jgrc24319-bib-0043] Zhang, J. , & Rothrock, D. A. (2005). The effect of sea‐ice rheology in numerical investigations of climate. Journal of Geophysical Research, 110, C08014. 10.1029/2004JC002599

